# Upcycling Pomegranate Peel Waste for Comparative Green Synthesis of Silver Nanoparticles: Characterization and Biological Applications

**DOI:** 10.1002/cbdv.71335

**Published:** 2026-05-12

**Authors:** Mandy Sibiya, Tshiamo B. Leta, Jerry O. Adeyemi, Stephen O. Amoo, Olaniyi A. Fawole

**Affiliations:** ^1^ South African Research Chairs Initiative in Sustainable Preservation and Agroprocessing Research, Faculty of Science University of Johannesburg Johannesburg South Africa; ^2^ Postharvest and Agroprocessing Research Centre, Department of Botany and Plant Biotechnology University of Johannesburg Johannesburg South Africa; ^3^ Vegetables, Industrial and Medicinal Plants Agricultural Research Council Pretoria South Africa; ^4^ Unit for Environmental Sciences and Management, Faculty of Natural and Agricultural Sciences North‐West University Potchefstroom South Africa

**Keywords:** antioxidant, food pathogen, green synthesis, silver nanoparticles, waste valorization

## Abstract

Pomegranate fruit peel was upcycled as a mediating agent for the comparative green synthesis of silver nanoparticles (AgNPs) utilizing facile (F), microwave (M), and autoclave (A) methodologies. XRD, SEM, TEM, and FTIR analyses revealed successful synthesis of AgNPs, with FTIR confirming polyphenol involvement in reduction and stabilization. The SEM micrographs indicated that all nanoparticles agglomerate into clusters. The TEM micrographs indicated that all nanoparticles are spherical, with varying particle sizes. In addition, the average particle size for AgNPs‐A was 17.84 nm, 14.91 nm for AgNPs‐F, and 14.23 nm for AgNPs‐M. Furthermore, the obtained XRD patterns showed crystallinity across all methods, with the microwave route producing the smallest crystallite size (6.58 nm) as estimated by the Scherrer equation. It is important to note that the crystallite size estimated from XRD represents the coherent diffraction domain and is not directly equivalent to the particle size observed by TEM. The antimicrobial property of AgNPs‐M was superior, especially against *Staphylococcus aureus* amongst other food‐borne pathogens, with an inhibition zone of 22.6 mm at 5 mg/mL. This study provides insights into three green synthesis methods for AgNPs using pomegranate peel waste, offering a foundation for future studies on safety, stability, and application‐oriented assessments.

## Graphical abstract

Pomegranate peel waste is upcycled for the green synthesis of silver nanoparticles using facile, microwave, and autoclave methods. The microwave approach produces smaller, crystalline nanoparticles with enhanced antioxidant and antimicrobial activity, particularly against food‐borne pathogens. The findings demonstrate the potential of fruit waste‐derived nanomaterials for sustainable antimicrobial applications.

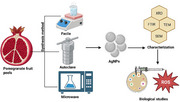

## 1 Introduction

The South African pomegranate (*Punica granatum)* industry has experienced significant growth over the past decade, driven by rising demand both domestically and internationally [1]. Consequently, production of the fruit reached approximately 12 984 tons in 2019 [1]. The pomegranate fruit is consumed globally in the form of fresh fruit, juice, or health supplements, attributed to its notable nutritional and medicinal benefits [2]. Notably, pomegranate juice is valued for its health benefits, driving steady expansion in the juice industry. However, this sector often regards the peel and seeds as waste [2]. The peel typically makes up approximately 40%–50% of the total fruit weight, which thus implies that nearly half of the harvested biomass becomes processing waste, contributing significantly to agro‐industrial waste streams if left not utilised [3]. The peel is abundant in bioactive compounds, including polysaccharides, phenolics, and flavonoids [3]. Therefore, this peel waste emerges as a promising candidate for valorisation, particularly in the green synthesis of nanoparticles, such as silver nanoparticles.

Nanoparticles are defined as small particles ranging from 1 to 100 nm, and their significance stems from their size and high surface area‐to‐volume ratio, which influences their physicochemical properties [4]. This technology has found applications across various fields, including food packaging, dietary supplements, textiles, and medicine [5]. Among these, silver nanoparticles are noted for their antibacterial activity, chemical stability, catalytic activity, and conductivity [6]. However, traditional physical and chemical synthesis methods for silver nanoparticles typically involve hazardous chemicals that are detrimental to human health and the environment, require significant energy consumption, and are often slow [7, 8]. Thus, green synthesis methods for nanoparticles have garnered attention due to their cost‐effectiveness and environmentally friendly nature [9]. Several literature reports have explored the use of plants, bacteria, and fungi for the biosynthesis of nanoparticles [10]. Specifically, various plant parts, including barks [11], fruits [12], leaves [13], and seeds [14], have been employed in the synthesis of silver nanoparticles. Approaches such as microwave‐assisted synthesis of silver nanoparticles [15] and autoclave‐assisted synthesis [16] have also been investigated. For instance, zinc oxide nanoparticles fabricated using the microwave and *Centella asiatica* (Linn.) leaf extract as a capping agent demonstrated impeccable antimicrobial activity against *Staphylococcus aureus* and *Escherichia coli* [17]. Furthermore, autoclave‐synthesised silver nanoparticles using *Canna indica* L. demonstrated remarkable antimicrobial activity against *E. coli*, *S. aureus*, and *Klebsiella pneumoniae* [16]. However, most studies focus on a single synthesis route, limiting direct comparisons across different green synthesis methods of silver nanoparticles.

While the synthesis of silver nanoparticles using plant‐mediated routes has been extensively explored over the years due to the broad‐spectrum of biological applications, catalytic functions, and its environmental sustainability attributes [18, 19], most of these studies either adopted the single synthesis route predominantly or used varying precursor compositions and reaction conditions. These approaches, while very useful, introduce variability that significantly limits the possibility of attributing observed physicochemical and biological properties to the synthesis pathway. For example, the differences in extract composition, reaction kinetics, and energy input mechanisms have been explored and found to influence morphology, particle size, and bioactivity [20, 21]. Consequently, existing comparative studies often provide only incremental insights, as they fail to isolate the intrinsic effects of synthesis routes under controlled chemical conditions. This limitation is largely attributed to the strong dependence of nanoparticle properties on multiple interacting parameters, such as precursor concentration, temperature, pH, and the nature of capping agents, which are often varied. To this end, some reviews have established that these parameters influence the morphology, size, stability, and functional performance, which in turn complicate the direct comparison between synthesis approaches when the conditions for the synthesis are not standardized [22, 23]. As a result, the absence of controlled comparative frameworks continues to obscure the true contribution of synthesis pathways, reinforcing the need for systematic investigations under identical chemical conditions. Therefore, there is a need for a systematic comparison of different synthetic pathways to better understand how processing conditions influence nanoparticle characteristics and to inform future optimization for controlled and scalable production.

Accordingly, this study aims to compare various green synthesis methods, including facile, autoclave, and microwave techniques, to produce silver nanoparticles utilizing pomegranate peel waste as a stabilizing and capping agent. Although many studies in the literature have been conducted synthesis independently, or in partial comparison using these routes, limited reports exist for the use of same precursor concentration and extract conditions. Thus, there is a need to explore the assessment of how these synthesis pathways influence nanoparticle characteristics (size, morphology, crystallinity, and stability). Additionally, the biological activity of the resulting nanoparticles, including antibacterial and antioxidant properties, was assessed. It is important to note that in this study, the temperature and duration employed for each synthesis route are reflective of the intrinsic operational characteristics of each method. While hydrothermal synthesis requires elevated temperature and prolonged reaction time, microwave and facile approaches involve rapid heating profiles. Based on this rationale, we hypothesized that the intrinsic energy delivery mechanism of each synthesis route would differentially influence nucleation–growth dynamics and consequently modulate nanoparticle size, crystallinity, and biological performance under otherwise identical chemical conditions. The study, therefore, evaluates route‐dependent effects under consistent precursor and pH conditions rather than identical thermal exposure.

## 2 Results and Discussion

### 2.1 FTIR‐Spectroscopy of the Extract and the Biosynthesis of AgNPs

**FIGURE 1:**
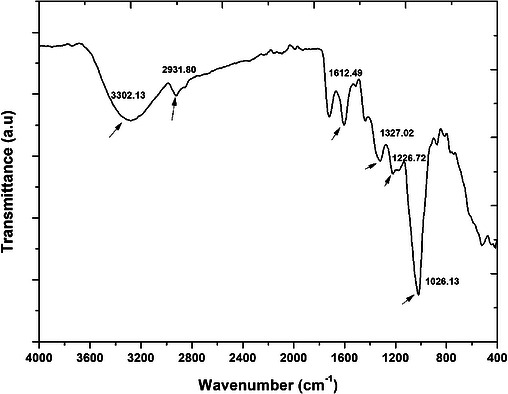
FTIR spectrum for the pomegranate peel powder before AgNPs synthesis.

The observed spectra of the peel extract are presented in Figure 1. Important functional groups that played a major role in mediating the synthetic process of AgNPs by stabilizing and capping the nanoparticles have been highlighted in the spectra. The peak at 3302 cm^−1^ corresponds to O─H stretching vibration, typically associated with hydroxyl groups from phenolic compounds [17]. This group is likely to participate in the reduction of Ag^+^ to AgNPs. Furthermore, the observed peak at about 2932 cm^−1^, related to the C─H stretching vibration, suggests the presence of aliphatic groups within the plant extract [24]. In addition, the peaks at 1613 and 1372 cm^−1^ are found within ranges commonly associated with aromatic/amide and C─N/phenolic vibrations in plant extracts; nevertheless, these assignments are indicative and not definitive without peak deconvolution or reference spectra [24]. Also, the peaks at 1227 and 1026 cm^−1^ are associated with the C═O vibrational, probably emerging from aromatic carbonyl or amide compounds [17]. Overall, all the observed stretching vibrations related to the observed functional group, including carbonyl, amides, and hydroxyl, were implicated in the mediating and stabilization properties of the plant extract, confirming the plant extract's role as a reducing agent [24]. These findings are in alignment with literature reports, where similar phytochemicals facilitate nanoparticle synthesis using Kei apple leaves and pomegranate peel extracts [2, 25].

**FIGURE 2:**
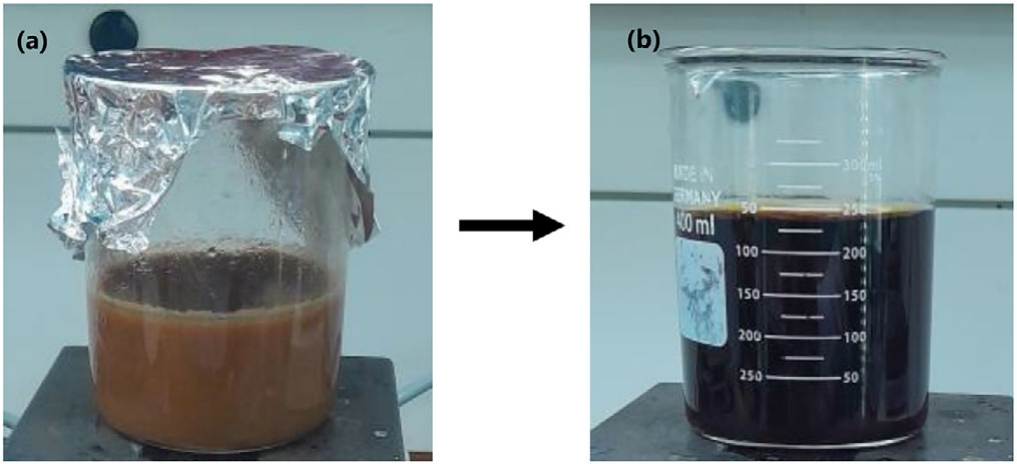
The photographic representation of the aqueous pomegranate peel extract (a) and pomegranate peel extract (PPE) with silver nitrate (b) depicts the colour change from brown‐black to black.

For the biosynthesis of AgNPs, a colour change from brown‐black to black was observed upon adding silver nitrate (AgNO_3_) in the aqueous pomegranate fruit peel extract for all synthesis methods (Figure 2). This can be utilised as the preliminary indication of the synthesis of AgNPs due to the excitation of surface plasmon vibration in the AgNPs, thus indicating the reduction of silver ions [26]. Similar results were observed by [27], in which a colour change from pale yellow to a brownish solution was observed after adding AgNO_3_. It is important to note that the study maintains consistent precursor concentration and pH while allowing method‐specific thermal and volume conditions to reflect intrinsic process characteristics. Thus, observed differences are attributed to route‐dependent nucleation–growth dynamics rather than experimental bias.

### 2.2 Comparative Analysis of XRD Study of AgNPs Synthesized Via Autoclave, Microwave, and Facile Methods

**FIGURE 3:**
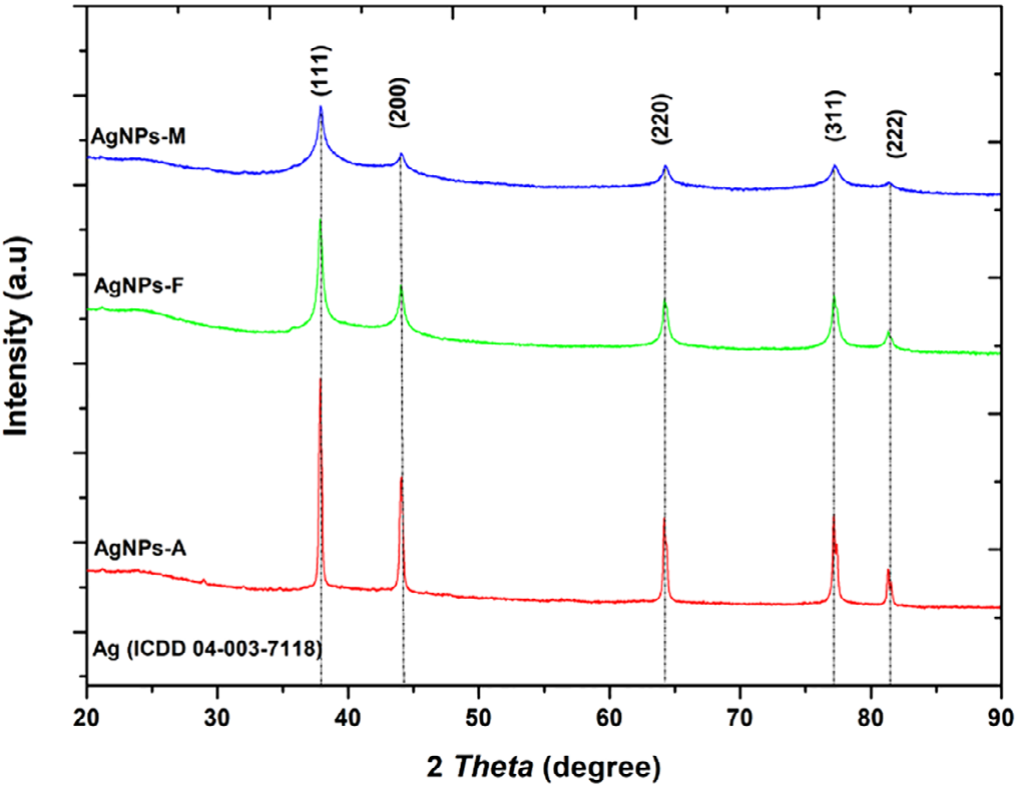
X‐Ray diffraction pattern for the synthesized pomegranate peel extract‐mediated AgNPs using microwave (AgNPs‐M), autoclave (AgNPs‐A), and facile (AgNPs‐F) approaches.

The diffractogram obtained from x‐ray diffraction (XRD) analysis of silver nanoparticles (AgNPs) prepared using the autoclave (A), microwave (M), and Facile (F) methods is presented in Figure 3. The observed peaks at 37.9°, 44.1°, 64.2°, and 77.2° were indexed as (111), (200), (220), and (311) planes, respectively, which correspond to the expected face‐centred cubic (FCC) crystalline structure of AgNPs [28] and JCPDS file no 04‐003‐7118 [29]. The absence of other peaks in all the methods used suggests that pomegranate peel extract (PPE) effectively serves as a good mediating agent, as shown in Figure 3. The peaks were sharp and intense for all the approaches used, suggesting a high degree of crystallinity in the AgNPs [30]. Comparing the mean crystalline size of the nanoparticles in the used approaches, Debye–Scherrer's Equation (1) was employed as follows:

(1) $$D=\frac{K\lambda }{\beta Cos\theta }$$

where *D* = crystallite size of AgNPs in nm, *K* = Scherrer's shape factor (0.90), λ = the x‐ray wavelength used (1.5406 Å), β = full width at half maximum (FWHM) of the (111), (200), (220), and (311) reflections, and the reported value represents the mean ± SD of the sizes obtained from these peaks, and it is given in radians, and θ = Bragg diffraction angle in degrees. Thus, the microwave method (AgNPs‐M) produced nanoparticles with an average size of 6.58 nm, whereas autoclave synthesis (AgNPs‐A) and facile synthesis (AgNPs‐F) yielded average particle sizes of 16.93 and 11.84 nm, respectively. The smaller size observed with the AgNPs‐M approach has been attributed to the rapid, uniform energy distribution in microwave processing [31]. Additionally, the observed smaller size has been documented to translate into a higher surface area‐to‐volume ratio, which, in turn, is advantageous in applications that demand high surface reactivity, such as biological treatment, as antimicrobial agents. On the other hand, the facile approach (F) yielded an intermediate particle size, demonstrating a balance between speed and temperature control, compared with the autoclave method (A), which produced the largest particles due to the high temperature and pressure over a prolonged period. Hence, even though these two other approaches seemed to possess bigger particle sizes compared to the microwave method (M), the intermediate size observed for F suggests the possibility for versatility, where a compromise in reactivity and stability is required, making them more adaptable. Whereas, for the particles synthesized using A, although larger, these particles may be useful where stability and bulk are prioritized, possibly in applications that require slow‐release or sustained activity. Overall, using green synthesis with PPE as a biogenic mediator demonstrated a successful route to pure and crystalline AgNPs production, as seen in other reports [29, 32].

### 2.3 Microscopic Study

**FIGURE 4:**
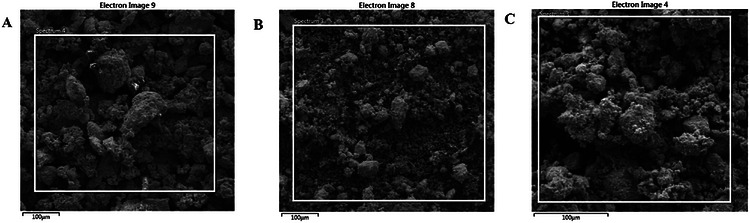
SEM micrograph of green synthesised Ag‐NPs whereby A represents AgNPs‐A, B AgNPs‐F, and C represents AgNPs‐M.

**FIGURE 5:**
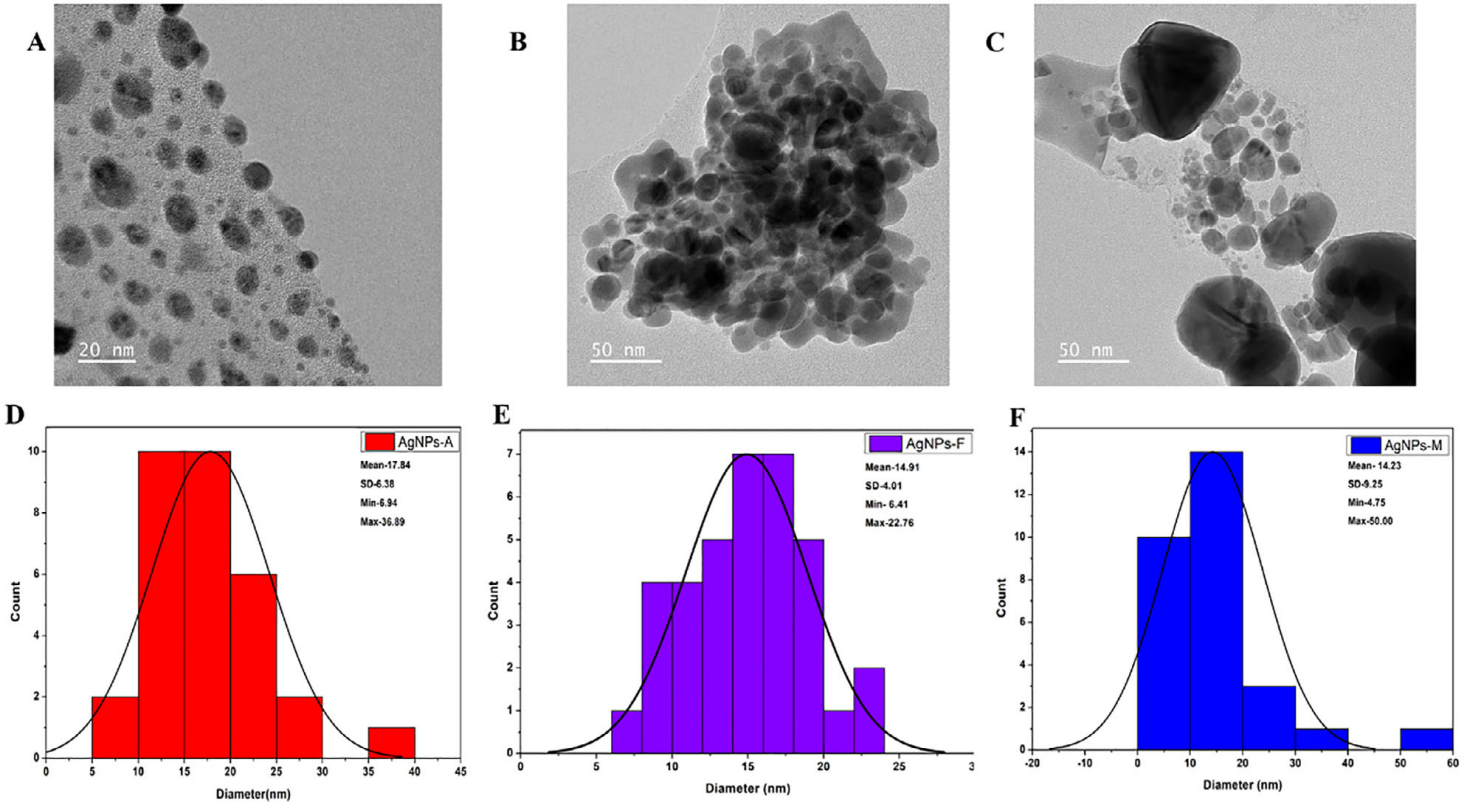
TEM micrograph of green synthesized Ag‐NPs whereby A represents AgNPs‐A, B AgNPs‐F, and C represents AgNPs‐M with the Histogram plot (D‐F) whereby D represents AgNPs‐A, E AgNPs‐F, and F represents AgNPs‐M.

The structural characteristics of silver nanoparticles (AgNPs) synthesized via various synthetic routes were analyzed using scanning electron microscopy (SEM) and transmission electron microscopy (TEM), as illustrated in Figure 4A–C. The SEM micrographs indicate that all synthesized nanoparticles tend to aggregate into clusters. This phenomenon may be attributed to the high surface energy of the nanoparticles and to insufficient phytochemical capping during plant‐mediated synthesis [33]. Additionally, the drying of the samples during SEM preparation may have further facilitated cluster formation, a common phenomenon in green‐synthesized nanoparticles lacking external stabilizers [33]. Conversely, the TEM images presented in Figure 5A–C revealed a predominantly spherical morphology with varying particle sizes. Specifically, the particle sizes of AgNPs‐A (Figure 5A,D) ranged from 6.94 to 36.89 nm, with an average size of 17.84 ± 6.38 nm. The nanoparticles were primarily spherical and moderately polydispersed, with some signs of aggregation, a phenomenon commonly observed in plant‐mediated synthesis systems due to differences in nucleation and growth dynamics under varying reaction conditions. The larger particle sizes observed may be attributed to the elevated temperature and pressure during nanoparticle synthesis, potentially altering their functionality [34]. Similarly, AgNPs‐F (Figure 5B,E) also displayed a predominantly spherical and moderately polydispersed morphology, albeit with some observable agglomeration. Their particle sizes ranged from 6.41 to 22.76 nm, with an average size of 14.91 ± 4.01 nm. This moderate particle size suggests a balance between reaction kinetics and temperature control during synthesis, which may enhance their versatility by providing a compromise between reactivity and stability, thereby making them more adaptable [35]. Furthermore, AgNPs‐M were also characterized as spherical and polydispersed with minimal agglomeration, exhibiting particle sizes ranging from 4.75 to 50.00 nm and an average size of 14.23 ± 9.25 nm. The relatively broad size distribution (4.75–50.00 nm) indicates a degree of polydispersity, which is typical of green synthesis approaches due to the heterogeneous nature of phytochemical‐mediated nucleation and growth. The relatively small particle size can be attributed to the rapid and uniform energy distribution achieved through microwave synthesis, resulting in a higher surface area‐to‐volume ratio, which contributes to enhanced functional properties such as antimicrobial and antioxidant activity [31]. While XRD analysis indicates smaller crystallite domains for AgNPs‐M, the TEM analysis reflects the overall particle size and morphology, which may consist of one or more crystallites; therefore, direct comparison between these measurements should be interpreted cautiously. To provide a more representative description of the nanoparticle population, statistical size distribution analysis based on TEM measurements has been included as histogram plots (Figure 5D–F), with corresponding average particle sizes and standard deviations reported. This trend is consistent with the smaller crystallite domains observed from x‐ray diffraction (XRD) analysis, although both techniques probe different structural scales. Therefore, the utilization of green synthesis employing pomegranate peel extract as a biotic material demonstrates a successful approach to the production of pure and crystalline AgNPs, consistent with findings from previous studies [29, 32].

### 2.4 Biological Study of the Synthesised Pomegranate Peel Extract‐Mediated AgNPs Using Microwave (AgNPs‐M), Autoclave (AgNPs‐A), and Facile (AgNPs‐F) Approaches

#### 2.4.1 Antimicrobial Evaluation

**FIGURE 6:**
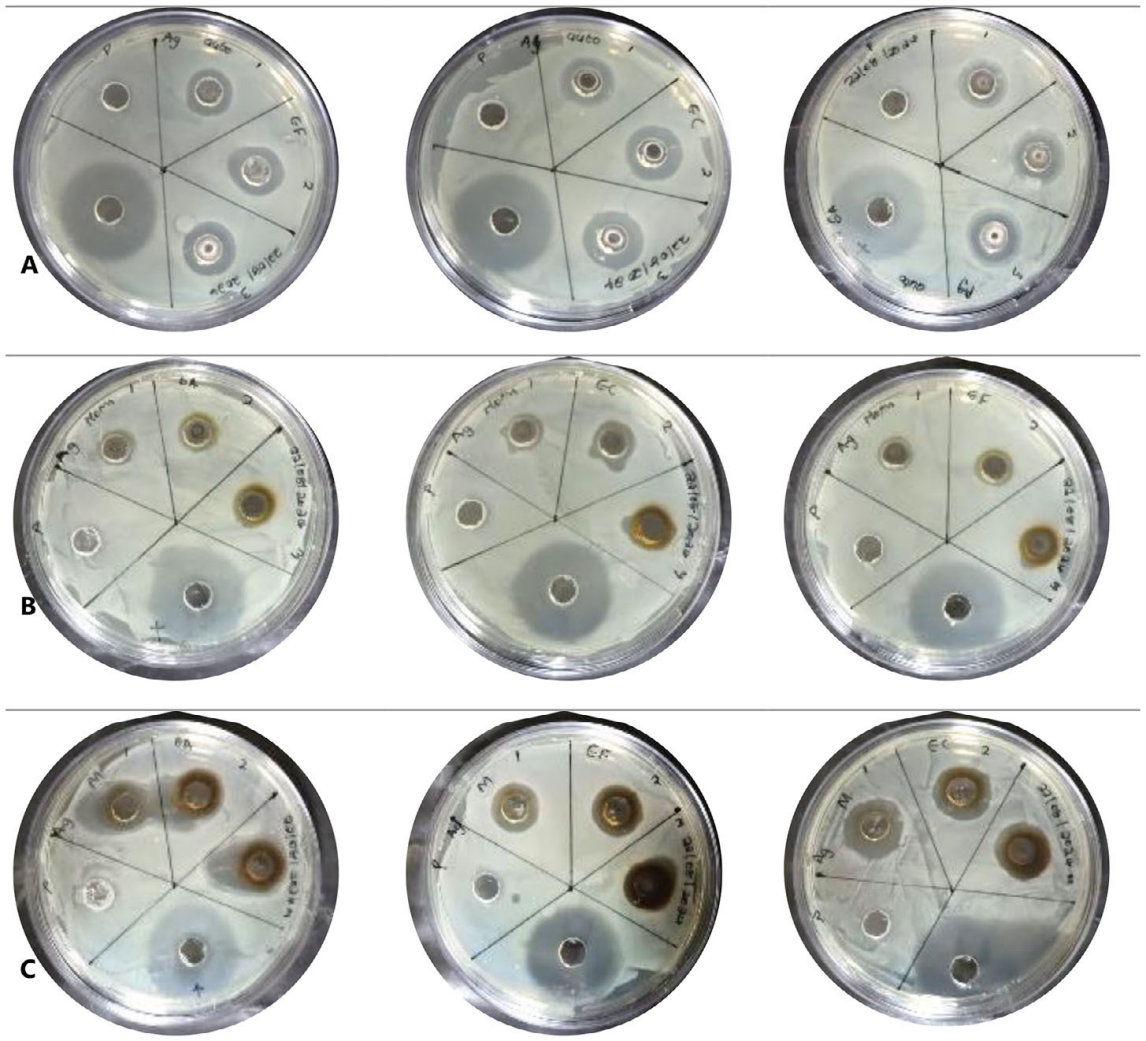
Pictorial representation of the inhibitory effect of silver nanoparticles synthesised through autoclave (A), facile (B) and microwave (C) on the MH‐agar plates with 1–10 mg/ mL, 2–5 mg/mL and 3–2.5 mg/mL and *p* denoted for distilled water as the negative control and the largest zone of inhibition demonstrates the positive control (tetracycline) against *E. coli* (EC), *S. aureus* (SA) and *E. faecalis* (EF).

The antimicrobial evaluation in this study aims to identify the most efficient method to enhance their biological properties for potential applications in food preservation and antimicrobial packaging. It is important to note that although the antimicrobial assessment was conducted using agar well diffusion as a comparative screening method, the results reflect relative performance among samples, with consistent conditions ensuring valid comparisons despite possible diffusion limitations. Generally, AgNPs have been well documented for their antimicrobial activity; thus, the inhibition of the synthesized silver nanoparticles is summarized in Table 1 and Figure 6. All nanoparticles were evaluated against pathogenic bacteria, namely, *E. coli (EC)*, *S. aureus (SA)*, and *Enterococcus faecalis (EF)*, with tetracycline (TC) as a reference control. It is important to note that tetracycline was included solely as a positive control to validate the antimicrobial assay and confirm the susceptibility of the test organisms, and not for direct equivalence comparison with the synthesized AgNPs. In the activity study against *SA*, AgNPs‐M showed the strongest inhibitory effect with a maximum inhibition zone of 22.6 ± 4.25 mm at 5 mg/mL, which is notably higher than the activity at 10 mg/mL (18 ± 1.52 mm), probably associated with the aggregation of the nanoparticles at higher concentrations, decreasing surface interaction. However, no direct aggregation measurements were conducted; therefore, this observation is interpreted cautiously as a possible concentration‐dependent variation in nanoparticle interaction. AgNPs‐A and AgNPs‐F followed with 15 ± 0.00 mm and 13 ± 2.51 mm at 10 mg/mL, respectively. Nevertheless, in all the synthetic approaches, the synthesized AgNPs showed lower activity compared to tetracycline (27.6 ± 0.84 mm). This comparison is presented only to provide context for antimicrobial efficacy and does not imply direct equivalence in mechanism or performance. Also, in the activity study against EC, a similar trend was observed, with better responses at lower concentrations improving bioavailability due to better dispersion, with AgNPs‐M exhibiting 18.6 ± 2.18 mm at 2.5 mg/mL and 16.3 ± 0.88 mm at 5 mg/mL. Whereas AgNPs‐A and AgNPs‐F demonstrated lower zones of inhibition (15–16.6 mm and 11–12 mm, respectively) at these concentrations. Overall, *E. coli* appeared more susceptible to AgNPs, consistent with previous reports noting the increased permeability of Gram‐negative bacterial membranes to silver ions [36]. And finally, the study against EF showed a weaker activity compared to the other strains, with inhibition zones ranging between 12–16 mm for AgNPs‐M, and between 11–16.6 mm for AgNPs‐A and AgNPs‐F, showing moderate activity. These results suggest that *E. faecalis*, a Gram‐positive bacterium with a thicker peptidoglycan wall, is relatively more resistant to AgNPs, likely due to limited silver ion penetration [14, 36]. Comparatively, microwave‐assisted AgNPs‐M generally had better antibacterial activity in all used concentrations and organisms, which can be attributed to the smaller particle size, leading to higher surface area and increased release of silver ions [14]. In addition, the observed variability among bacterial species may be associated with cell wall structural differences, with Gram‐negative bacteria (*E. coli*) having a thinner peptidoglycan layer and porins, which allowed for easier ion diffusion, while Gram‐positive bacteria (*S. aureus* and *E. faecalis*) exhibit greater resistance [36]. On the other hand, the relatively lower activity observed in AgNPs‐F may be associated with the observed particle size and less efficient surface capping, which may be likely due to prolonged thermal treatment during synthesis, degrading the bioactive capping agents from the pomegranate extract. Overall, these findings underscore the role of synthesis approaches in tailoring the bioactivities of green‐synthesized silver nanoparticles. Specifically, the microwave‐assisted approach offered a promising synthetic method for preparing biologically active AgNPs with broad‐spectrum antibacterial properties, supporting their application as active additives for food packaging materials, aligning with previous literature [32, 37, 38].

**TABLE 1:** The antibacterial activity of the synthesized nanoparticles (AgNPs‐M^a^, AgNPs‐A^b^, and AgNPs‐F^c^) and Tetracycline (TC) against *S. aureus* (SA), *E. coli* (EC), and *E. faecalis* (EF).

	Zone of inhibition(mm)									
	10 mg/mL			5 mg/mL			2.5 mg/mL			10 mg/mL
Sample codes	AgNPs‐M	AgNPs‐A	AgNPs‐F	AgNPs‐M	AgNPs‐A	AgNPs‐F	AgNPs‐M	AgNPs‐A	AgNPs‐F	TC^e^
*SA* (ATCC25428)	18 ± 1.52^b^	15 ± 0.00^b^	13 ± 2.51^b^	22.6 ± 4.25^a^, ^b^	16.6 ± 0.33^b^	14 ± 1.52^b^	17 ± 2.64^b^	16.3 ± 0.33^b^	11.3 ± 0.88^c^	27.6 ± 0.84^a^
*EC* (ATCC25922)	18 ± 1.15^b^	15 ± 0.00^b^	11 ± 0.57^c^	16.3 ± 0.88^b^	15.6 ± 0.33^b^	11 ± 0.57^c^	18.6 ± 2.18^b^	16.6 ± 0.33^b^	12 ± 0.00^c^	29.4 ± 0.37^a^
*EF* (ATCC2921)	16 ± 0.00^b^	14.3 ± 0.66^b^, ^c^	11 ± 0.57^c^	16 ± 0.57^b^	15.6 ± 0.33^b^	11 ± 0.00^c^	12 ± 0.00^c^	16 ± 0.57^b^	12.3 ± 0.33^c^	29.4 ± 0.41^a^

*Note*: Average values and their respective ± standard deviation (*n* = 3).

^a^ Numerical data with distinct letters indicate significant differences in their respective column conducted using Duncan's multiple range test (*p* < 0.05).

^b^ AgNPs‐M Silver nanoparticles synthesized through microwave.

^c^ AgNPs‐A Silver nanoparticles are synthesized through autoclaving.

^d^ AgNPs‐F Silver nanoparticles are synthesized through a facile method.

^e^ Tetracycline has the positive control. TC = Tetracycline

### 2.5 Antioxidant Study of the Synthesized Pomegranate Peel Extract‐Mediated AgNPs Using Microwave (AgNPs‐M), Autoclave (AgNPs‐A), and Facile (AgNPs‐F) Approaches Using ABTS and DPPH

Generally, antioxidant activity plays a key role in understanding the food‐packaging and biomedical, and potential of nanoparticles. Thus, over the years, methods such as ABTS and DPPH assays have been employed to measure the radical scavenging potential of various materials, which serve as indicators of antioxidant potential, with higher percentages indicating better radical scavenging capabilities. In addition, the minimum concentration required to inhibit 50% of radicals (IC_50_) provides a measure of potency, with lower IC_50_ values indicating higher effectiveness [39]. The observed data from both studies, alongside the estimated IC_50_ for AgNPs synthesized by microwave (AgNPs‐M), autoclave (AgNPs‐A), and facile (AgNPs‐F) methods, and the standard ascorbic acid (AA) obtained in the ABTS assay, have been collected and summarized in Table 2. The ABTS radical cation decolorization assay, measured spectrophotometrically at 734 nm [33], showed that all samples exhibited notable antioxidant activity, though with varying intensities. Ascorbic acid (AA), used as the standard, showed the highest radical scavenging across all concentrations, with an IC_50_ value of 1.048 mg/mL. Amongst the synthesized nanoparticles, AgNPs‐M showed the strongest antioxidant activity, with a peak of 83.88% at 0.025 mg/mL and an IC_50_ of 1.126 mg/mL, closely matching the standard. Whereas AgNPs‐A and AgNPs‐F showed moderate activity. The superior performance of AgNPs‐M relative to its counterparts is likely due to the small particle size and larger surface area produced by microwave synthesis, which enhances the nanoparticles' electron‐donating ability. It is important to note that potential assay interference was accounted for using nanoparticle blanks and baseline corrections, as described in the methodology, thereby ensuring that the observed trends reflect intrinsic antioxidant behavior. Although some non‐monotonic trends were observed (e.g., AgNPs‐F at higher concentrations), no direct aggregation or dispersion stability measurements were conducted; therefore, no definitive mechanistic attribution is made. The observed behavior is cautiously interpreted as a concentration‐dependent variation in nanoparticle–radical interactions, and this limitation has been explicitly acknowledged. This observation agrees with other studies, which indicate that microwave‐assisted nanoparticles yield small nanoparticles with an enhanced ability to interact with and neutralize radicals [40]. Overall, AgNPs‐M displayed better radical scavenging activity than AgNPs‐A and AgNPs‐F.

**TABLE 2:** Radical scavenging activity (%) and the IC_50_ values (mg/ml) of ascorbic acid (AA), AgNPs‐M, AgNPs‐A and AgNPs‐F for ABTS.

Sample code	Radical scavenging activity (%) for ABTS^a^				
Conc. (mg/mL)	0.00625	0.0125	0.025	0.05	IC_50_ f
AA^b^	83.02 ± 0.687	96.50 ± 0.388	95.69 ± 0.198	97.55 ± 0.750	1.048
AgNPs‐M^c^	56.18 ± 4.433	78.47 ± 0.721	83.88 ± 0.276	69.57 ± 1.877	1.126
AgNPs‐A^d^	23.21 ± 1.354	33.11 ± 7.418	60.99 ± 0.327	63.31 ± 2.788	1.523
AgNPs‐F^e^	65.30 ± 3.851	89.92 ± 0.208	80.97 ± 2.500	68.53 ± 2.478	1.153

Average values and their respective ± standard deviation (*n* = 4).

^a^ 2,2'‐azino‐bis(3‐ethylbenzothiazoline‐6‐sulfonic acid).

^b^ Ascorbic Acid.

^c^ Silver nanoparticles synthesized through microwave.

^d^ Silver nanoparticles are synthesized through an autoclave.

^e^ Silver nanoparticles are synthesized through a facile method.

^f^ 50% inhibition concentration.

Similarly, in the DPPH assay (Table 3), the standard AA showed a concentration‐dependent scavenging profile, increasing from 3.80% at 0.00625 mg/mL to 94.19% at 0.05 mg/mL, with an IC_50_ of 2.79 mg/mL. Notably, all AgNP samples showed lower IC_50_ values than AA, suggesting stronger radical neutralization at lower concentrations. Specifically, AgNPs‐M showed the strongest and most consistent activity (55.33%–61.77%), with an IC_50_ of 1.111 mg/mL, while AgNPs‐A closely followed with moderate activity and an IC_50_ of 2.073 mg/mL. In contrast, AgNPs‐F showed a non‐monotonic trend, with higher inhibition at lower concentration (73.13%) followed by a gradual decrease as concentration increased, yielding an IC_50_ of 2.175 mg/mL. Although an IC_50_ value was calculated for AgNPs‐F, the nonlinear dose–response pattern suggests that this value should be interpreted cautiously. This unusual behaviour observed for AgNPs‐F may be influenced by changes in nanoparticle dispersion behavior; however, aggregation was not measured in this study. Generally, compared with ascorbic acid (AA), a strong antioxidant, all nanoparticles showed better scavenging activity. Overall and comparatively, within the observed activity pattern among the prepared silver nanoparticles, AgNPs‐M gave the highest and most stable antioxidant potential, as indicated by both its consistent radical scavenging percentages and its low IC_50_ value, while AgNPs‐A exhibited moderate activity, especially at higher concentrations, and AgNPs‐F had high initial activity that decreases with concentration. This observation thus highlights that microwave synthesis may yield AgNPs with superior antioxidant properties, making them useful for applications that require stable and potent antioxidants.

**TABLE 3:** Radical scavenging activity (%) and the IC_50_ values (mg/ mL) of ascorbic acid (AA), AgNPs‐M, AgNPs‐A, and AgNPs‐F for DPPH.

Sample code	Radical scavenging activity (%) for DPPH^a^				
Conc.(mg/mL)	0.00625	0.0125	0.025	0.05	IC_50_ ^f^
AA^b^	3.80 ± 0.936	10.70 ± 4.525	38.49 ± 1.116	94.19 ± 0.193	2.79
AgNPs‐M^c^	55.33 ± 8.841	61.77 ± 7.702	52.45 ± 3.970	55.04 ± 2.098	1.111
AgNPs‐A^d^	23.70 ± 0.882	33.65 ± 2.706	55.28 ± 8.305	74.90 ± 2.355	2.073
AgNPs‐F^e^	73.13 ± 1.529	70.19 ± 3.467	64.37 ± 3.161	57.74 ± 2.177	2.175

*Note*: Average values and their respective ± standard deviation (*n* = 4).

^a^ 2,2‐diphenyl‐1‐picrylhydrazyl.

^b^ Ascorbic Acid.

^c^ Silver nanoparticles synthesized through microwave.

^d^ Silver nanoparticles are synthesized through autoclave.

^e^ Silver nanoparticles are synthesized through a facile method.

^f^ 50% inhibition concentration.

## 3 Conclusions

In conclusion, this study successfully demonstrated three green synthesis routes, involving the facile, autoclave, and microwave approaches for silver nanoparticles (AgNPs) using pomegranate peel waste as a mediating agent under typical precursor and pH conditions. Structural characterization using XRD indicated the formation of crystalline AgNPs across all methods, with observed variations in the estimated particle size and distribution, and the microwave method yielded comparatively smaller sizes. The prepared nanoparticles demonstrated measurable antibacterial and antioxidant activities in a preliminary study. Specifically, in the antimicrobial activity study against common food pathogens, all synthesized nanoparticles exhibited useful activity, underscoring the potential of silver nanoparticles (AgNPs) as antimicrobial agents in the design of active food packaging materials. Again, AgNPs‐M demonstrated greater antimicrobial activity than the facile and autoclave‐assisted synthesis methods, attributed to its smaller particle size under the tested conditions. Furthermore, in both ABTS and DPPH assays, AgNPs‐M exhibited better antioxidant properties, which were slightly lower than those of ascorbic acid, as indicated by the estimated IC_50_. Although the findings provide comparative insight into how synthesis routes influence nanoparticle characteristics and preliminary biological performance, further investigations, such as cytotoxicity assessment and detailed antimicrobial screening, are required before establishing applicability in food packaging systems. Nevertheless, overall, this work offers a platform for the comparison of green synthesis pathways using identical precursors and extract conditions, providing a foundational understanding of route‐dependent effects on AgNP properties while highlighting areas for further optimization and validation.

## 4 Experimental Section

### 4.1 Materials

All chemicals were used without further purification. Silver nitrate (AgNO_3_) was procured from Glass World, South Africa, while sodium hydroxide (NaOH) was sourced from Rochelle Chemicals, South Africa. Additional reagents, including 2,2‐azino‐bis[3‐ethylbenzothiazoline‐6‐sulphonic acid] (ABTS^+^), ascorbic acid (AA), absolute methanol, Mueller‐Hinton (MH) agar, and potassium persulfate, were obtained from Sigma–Aldrich, South Africa.

### 4.2 Preparation of Aqueous Peel Extract of *P. granatum*

**FIGURE 7:**
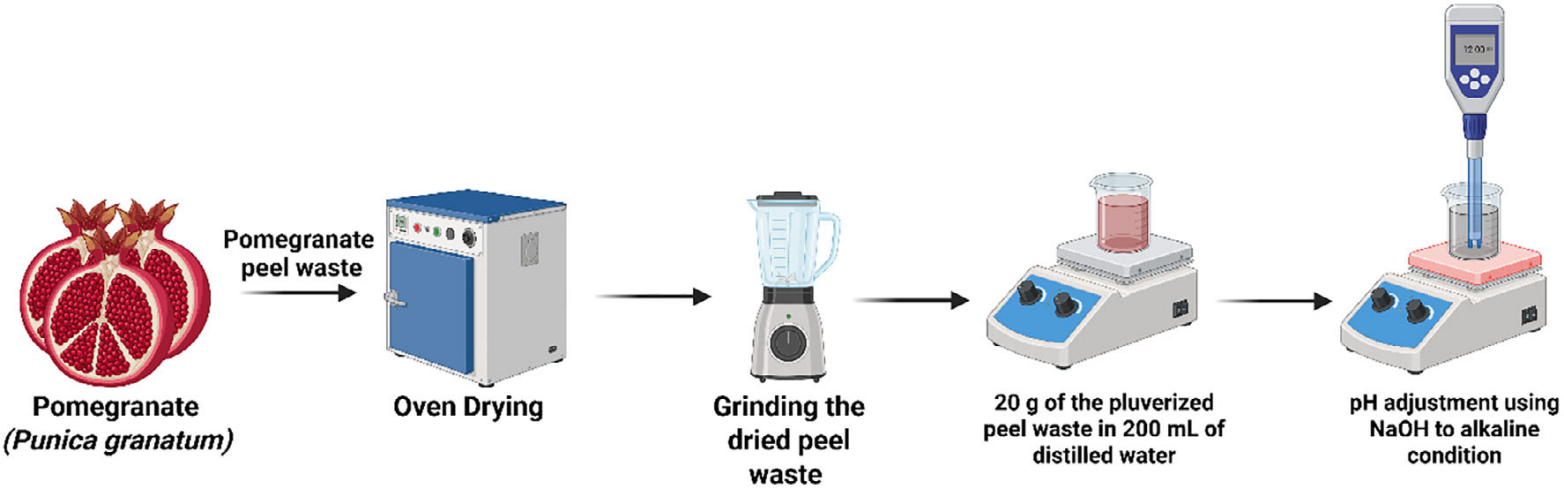
Schematic illustration of the extraction process of pomegranate fruit peel extract. Created in Bioreader.com.

*P. granatum* fruit peels were collected from Ubali Pomegranate Farm, Kameelfontein, Johannesburg, during the 2022 season. The peels were transported to the South African Research Chairs Initiative in Sustainable Preservation and Agroprocessing Research, Faculty of Science, University of Johannesburg, South Africa. Following collection, the fruit peels were cleaned with deionized water and disinfected with 0.01% sodium hypochlorite (NaClO). Subsequently, as shown in Figure 7, they were oven‐dried at 50°C for 3 days and ground into a powder. To prepare the extract, 20 g of pomegranate fruit peel powder was mixed with 200 mL of distilled water (dH_2_O) and heated at 50°C for 2 h. The resultant extract was filtered using Whatman No. 1 filter paper and stored in a refrigerator for further use. For subsequent use as a mediating agent for the AgNPs synthesis, in the different approaches used, the reaction pH was adjusted to 12 to promote deprotonation of phenolic constituents in the peel extract, as reported in the literature [41], which in turn enhanced their reducing properties toward silver ions, as commonly reported in alkaline‐mediated green synthesis systems. Maintaining this pH across all synthetic routes provided a platform for comparing processing effects.

### 4.3 Synthesis of Silver Nanoparticles (AgNPs) Using Aqueous *P. granatum* Fruit Peel Extract

#### 4.3.1 Facile Synthesis

**FIGURE 8:**
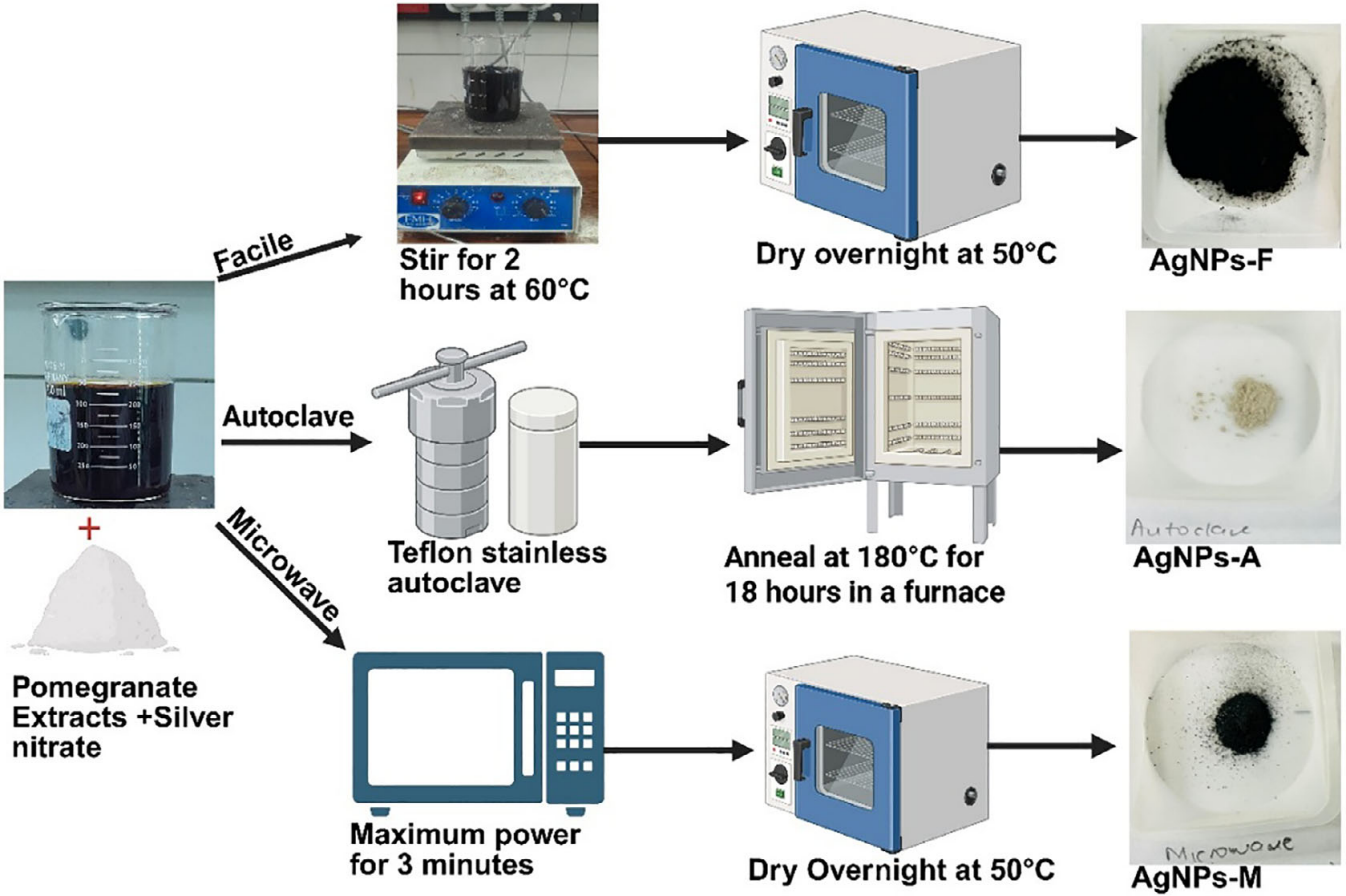
Schematic illustration of the synthesis of silver nanoparticles from pomegranate peel extract. Created in Bioreader.com.

The synthesis of silver nanoparticles (AgNPs) was conducted following the methodology outlined by Batterjee et al. [42], with modifications as illustrated in Figure 8. The obtained peel extract (200 mL) was adjusted to pH 12 with sodium hydroxide (NaOH). This peel extract was heated with a 1 mM solution of silver nitrate (AgNO_3_) (100 mL) at 60°C for 2 h until a brown‐black mixture was observed. The resultant mixture was centrifuged at 6,000 rpm for 15 min, and the pellet was washed with distilled water and 100% methanol. The collected pellet was then dried in an oven at 50°C overnight (12 h), subsequently ground into a fine powder, and designated as facile synthesized AgNPs (AgNPs‐F). The observed yield was 9.12 mg (84.44%).

#### 4.3.2 Microwave Synthesis

The synthesis of AgNPs was performed according to the procedure described by Ahammed et al. [43], with slight adaptations as illustrated in Figure 8. A 200 mL peel extract under alkaline conditions (pH 12) was combined with a 1 mM solution of silver nitrate (AgNO3) (100 mL) and exposed to microwave irradiation in an open system (Hisense Microwave oven, 50 Hz, China) for 3 min at 900 Watts until a brown‐black mixture was produced. The mixture underwent centrifugation at 6,000 rpm for 15 min, and the pellet was washed with distilled water and 100% methanol. The resulting pellet was dried in an oven at 50°C overnight (12 h), then ground into a fine powder and designated as microwave‐synthesized AgNPs (AgNPs‐M). The observed yield was 9.72 mg (90%).

#### 4.3.3 Autoclave Synthesis

The synthesis of AgNPs adhered to the methodology outlined by Shao et al. [44], with slight modifications as illustrated in Figure 8. A 50 mL solution of pomegranate peel extract, under alkaline conditions (pH 12), was combined with a 1 mM solution of silver nitrate (AgNO3) (50 mL) and placed in a Teflon‐lined stainless‐steel autoclave. The system was maintained at 180°C for 18 h in a furnace. Following this period, the system was allowed to cool to room temperature, after which the sedimented particles were dried overnight (12 h) at 50°C. The observed yield was 6.57 mg (60.83%).

Although total reaction volumes differed for the autoclave due to equipment configuration, identical precursor concentrations, extract‐to‐silver ratios, and pH conditions were maintained across all synthesis routes to ensure stoichiometric comparability.

### 4.4 Characterization of the Synthesized Pomegranate Peel Extract‐Mediated AgNPs Using Microwave (AgNPs‐M), Autoclave (AgNPs‐A), and Facile (AgNPs‐F) Approaches

X‐Ray diffraction (XRD) was employed to investigate the crystalline structure of the synthesized AgNPs. The XRD data were recorded in the range of 10° < 2θ < 80° at room temperature, with a scanning rate of 2°min^−1^, utilizing CuKα radiation (λ = 1.54060 Å) via an X‐ray diffractometer system (XPERT‐PRO). Instrumental broadening was not corrected in each approach; therefore, the reported values should be considered approximate and primarily useful for comparative assessment. The presence of functional groups within the peel extract responsible for capping and stabilizing AgNPs was identified using Fourier Transform Infrared Spectroscopy (FTIR, FR/R–4100 type A) across a spectral range of 4000–400 cm^−1^. The morphology of the AgNPs was examined utilizing a Scanning Electron Microscope (SEM) (SEM, SU8010, Hitachi, Japan) at 10 kV and subsequently analyzed through Transmission Electron Microscopy (TEM) (JEM‐2100) at 200 kV to ascertain the size and shape. Centrifugation of the nanoparticles was performed using a Heraeus Kendro Biofuge Stratos centrifuge, equipped with a fixed‐angle rotor (8 × 50 mL tube capacity, ∼34° angle). The rotor has a maximum radius of approximately 10–11 cm, consistent with standard high‐speed angle rotors for this system. Under these conditions, centrifugation at 6,000 rpm corresponds to an estimated relative centrifugal force (RCF) of ∼3,400–4,000 × g, calculated using the standard RCF equation.

### 4.5 Biological Studies of the Synthesized Pomegranate Peel Extract‐Mediated AgNPs Using Microwave (AgNPs‐M), Autoclave (AgNPs‐A), and Facile (AgNPs‐F) Approaches

The clinical isolates of bacterial strains, listed in Table 4, were obtained from the Department of Food Science at the University of Stellenbosch, South Africa, and were maintained on nutrient agar at 4°C for 24 h. The saturated cultures were diluted and adjusted with MH broth until an absorbance reading (at 600 nm) of 0.400–0.600 was achieved.

**TABLE 4:** List of foodborne pathogens used to evaluate the antibacterial activity of synthesized silver nanoparticles.

Gram stain	Species name	Strain number
Gram negative	*E. coli*	ATCC^a^ 25922
Gram positive	*E. faecalis*	ATCC 29212
	*S. aureus*	ATCC 25923

^a^ American Type Culture Collection.

### 4.6 Antibacterial Activity

The antibacterial activity of silver nanoparticles (AgNPs) was assessed using the disc well diffusion method as described by Abdelmigid et al. [45], with minor modifications. The study utilized common foodborne pathogens listed in Table 4 and employed Mueller‐Hinton agar plates for bacterial culture inoculation, which was performed using a sterile loop to ensure uniform distribution. Various concentrations of synthesized silver nanoparticles (10, 5, and 2.5 mg/mL) were introduced (50 µL) into each well (8 mm). The plates were incubated at 37°C for 24 h. Following incubation, the diameters of the clear zones of inhibition were measured in millimetres. Tetracycline, a standard antibiotic, was used as the positive control. Although tetracycline was used as a reference antibiotic control for comparative screening, direct potency equivalence is not implied.

### 4.7 Antioxidant Capacity

#### 4.7.1 DPPH Free Radical Scavenging Activity

The DPPH assay was conducted based on the protocols outlined by Swamy et al. [30] and Leta et al. [2], with slight modifications. A stock solution of silver nanoparticles was prepared at a concentration of 10 mg/mL in distilled water and then serially diluted to concentrations of 5, 2.5, 1.25, and 0.625 mg/mL. Subsequently, 15 µL of each solution was diluted in 100% methanol (735 µL) within glass cuvettes, followed by the addition of a methanolic DPPH solution (750 µL). Ascorbic acid was employed as the standard in the same concentration range as the silver nanoparticles. The samples were incubated for 30 min in the dark. After incubation, absorbance values were recorded using a UV‐visible spectrophotometer at a wavelength of 517 nm, with the spectrophotometer blanked against 100% methanol. Each sample was analysed in quadruplicate. The half maximal inhibitory concentration (IC_50_) for the samples was calculated using the standard formula presented in Equation (2):

(2) $$\begin{array}{ccc} \mathrm{DPPH}\;\mathrm{Scavenging}\;\mathrm{Activity}\;\left(\%\right) & = & \frac{Abs\;DPPH-Abs\;DPPH/Sample}{Abs\;DPPH}\\ & & \times \;100 \end{array}$$

where, Abs DPPH refers to the absorbance reading for methanolic DPPH without the nanoparticles or ascorbic acid, and Abs DPPH/Sample represents the absorbance reading for the control solution with the nanoparticles or ascorbic acid.

#### 4.7.2 ABTS^+^ Free Radical Scavenging Activity

The ABTS assay was performed in accordance with the methodologies outlined by Van Den Berg et al. [46]and Leta et al. [2], with slight modifications. A stock solution of silver nanoparticles was prepared at a concentration of 10 mg/mL in distilled water and subsequently serially diluted to concentrations of 5, 2.5, 1.25, and 0.625 mg/mL. Following this, 75 µL of each solution was further diluted with ABTS stock solution (1425 µL) in a glass cuvette. Ascorbic acid was again utilized as the standard in the same concentration range as the silver nanoparticles. The samples were incubated for 7 min in the dark. After incubation, absorbance values were again measured using a UV‐visible spectrophotometer at a wavelength of 734 nm, with the spectrophotometer blanked with 100% methanol. Each sample was recorded in quadruplicate. The half maximal inhibitory concentration (IC_50_) for the samples was similarly determined using the standard formula presented in Equation (3):

(3) $$\begin{array}{ccc} \mathrm{ABTS}\;\mathrm{Scavenging}\;\mathrm{Activity}\;\left(\%\right) & = & \frac{Abs\;ABTS-Abs\;ABTS/Sample}{Abs\;ABTS}\\ & & \times \;100 \end{array}$$

where, Abs ABTS denotes the absorbance reading for methanolic ABTS without the nanoparticles or ascorbic acid, and Abs ABTS/Sample indicates the absorbance reading for the control solution with the nanoparticles or ascorbic acid.

To ensure the reliability of the antioxidant assays and exclude possible interference, appropriate nanoparticle blanks were included and subtracted from sample readings to correct for background absorbance and turbidity effects. All measurements were performed in replicates (*n* = 3) with low standard deviations, confirming good reproducibility. Furthermore, the characteristic ABTS absorbance at 734 nm was monitored, with no observable spectral distortion, indicating minimal optical interference from the nanoparticles.

### 4.8 Statistical Analysis

The collected data were presented as mean ± standard deviation and analysed using one‐way ANOVA with Statistica software (Statistica 14.0, TIBCO, Tulsa, OK, United States of America). Duncan's multiple range test was employed following a significant ANOVA to compare group means at *p* < 0.05. Graphical representations were generated using Microsoft Excel, BioRender, and OriginPro.

## Author Contributions

**Mandy Sibiya**: writing – original draft, investigation, methodology, data curation. **Tshiamo B. Leta**: contributed to data collection and reviewed the manuscript. **Jerry O. Adeyemi**: supervised and coordinated the nanotechnology aspect of the work and reviewed the manuscript. **Stephen O. Amoo**: reviewed the manuscript and supervised **Olaniyi A. Fawole**: conceptualized, resources, project administration, reviewed the manuscript, and financed the research.

## Conflicts of Interest

The authors declare no conflict of interest regarding the publication of this article. All funding sources supporting the research work are acknowledged within the manuscript, and there are no financial, personal, or professional relationships that could be perceived as influencing the work reported in this article. The authors confirm that the research was conducted independently and in accordance with the ethical guidelines of the RSC.

## Data Availability

The data that support the findings of this study are available from the corresponding author upon reasonable request.
